# A novel scoring system proposal to guide treatment of dogs with hepatoid gland tumors

**DOI:** 10.3389/fvets.2025.1451510

**Published:** 2025-02-05

**Authors:** Lorella Maniscalco, Matteo Olimpo, Lorenza Parisi, Paolo Buracco, Eugenio Mazzone, Greta Martinelli, Marina Martano, Selina Iussich, Emanuela Morello

**Affiliations:** ^1^Department of Veterinary Sciences, University of Turin, Grugliasco, Italy; ^2^Department of Veterinary Medical Sciences, University of Parma, Parma, Italy

**Keywords:** dogs, neoplasm grading system, Ki67 antigen, perianal glands tumor, hepatoid tumors

## Abstract

Hepatoid perianal gland tumors are relatively common in dogs, accounting for 25% of all skin tumors. However, the specific factors involved in their development are still not completely clear. It has been established that hormonal influences can impact the formation of these tumors. The prognosis for dogs with perianal tumors depends largely on histology (benign vs. malignant) and, in case of malignancy, it has been suggested that the stage of the disease is important, with a more favorable outcome in dogs having small (under 5 cm in diameter), *non*-metastatic adenocarcinomas which are surgically removed with non-infiltrated margins. Nevertheless, there is a paucity of studies which thoroughly relate hepatoid gland histotypes to their prognosis; therefore, it is possible that a well-differentiated adenocarcinoma could be misclassified. Based on a retrospective review of 76 dogs with hepatoid gland tumors having clinical follow-up, the aims of this study were (1) to establish a histological grading system capable of potentially predicting prognosis and (2) to explore the role of Ki67 as a potential prognostic marker. Based on histopathological features only, the proposed grading system effectively differentiated tumors with a favorable prognosis from those with a worse prognosis to support histological diagnosis. The evaluation of the Ki67 index was not useful to predict prognosis in this study.

## 1 Introduction

Tumors of the perianal/hepatoid glands are quite prevalent in dogs, constituting 25% of all skin tumors (1). Hepatoid gland adenomas account for most canine perianal tumors in dogs (58–96%), while adenocarcinoma is rare (3–21%) (2–4). An influence of sexual hormones on the growth of adenomas, and even of differentiated adenocarcinomas, has been shown (5, 6). Adenoma occurs more frequently in intact male dogs (3, 7). In particular, intact male dogs over 10 years of age are 5.6 times more likely to develop hepatoid gland tumors than neutered male and spayed female dogs (6, 8).

The mechanisms involved in the development of hepatoid tumors are not completely understood (8–10).

The prognosis of malignant hepatoid perianal tumors, apart from histological features, depends on the clinical stage of the disease [TNM; (11)]. In fact, it has been reported to be favorable in dogs with a minimally invasive adenocarcinoma, less than 5 cm in diameter and without regional metastasis at presentation (12); additionally, it has also been suggested that prognosis may be influenced by the excision margin status (13). The prognosis for benign hepatoid perianal tumors is favorable, and castration and marginal surgical excision are curative, being the local recurrence rate very low; although it has been reported that an incomplete surgical resection may be a risk factor for recurrence (2, 14). Local regrowth after surgery in a neutered male is suggestive of tumor malignancy (3).

In the case of hepatoid malignancy, clinical stage (11) is the only prognostic factor identified; dogs bearing tumors larger than 5 cm in diameter have an 11-fold higher risk of death (12). According to the 1990 study byVail et al., dogs with stage 1 and stage 2 tumors had a disease-free interval of 2 years while a 1-year survival rate of 75% was recorded in dogs with T1 tumors and of 60% in dogs with T2 tumors. Median survival time for stage 3 and 4 was 6–12.5 months (12). Metastases are rare and are mostly found at the level of the sublumbar lymph nodes (15%); however, they can also occur in the lungs, liver, and spleen (12).

Regarding the histological morphology of perianal adenomas and epitheliomas, the most recent classifications agree that both entities have little or no nuclear atypia (2, 15). However, in contrast to epitheliomas, perianal adenomas usually consist of ordered lobules of well-differentiated hepatoid cells which form islands, cords and trabeculae. Adenocarcinomas are composed of disorganized, poorly differentiated, hepatoid cells with pleomorphic nuclei, prominent nucleoli, and vacuolated cytoplasm, having an elevated degree of nuclear atypia and an elevated mitotic rate in both the hepatoid and the reserve cell population (2, 15, 16). Thus, while the older textbooks considered the possibility of adenocarcinomas having a different degree of differentiation (17), the most recent classifications incorporate them into a single category characterized by frank malignant criteria (15). Well-differentiated adenocarcinomas were previously differentiated from adenomas by the presence of signs of tissue invasion, and a crowded or sloppy architecture (17); however, they are no longer mentioned in the new reference texts (15).

There are only a few prognostic immunohistochemical studies including Ki67 (14), COX-2 (15) and p53 (18). Adenocarcinomas showed a higher proliferation rate as compared to epitheliomas and adenomas grouped together, and a higher Ki-67 index was related to recurrence of perianal gland adenocarcinoma (14).

The aims of this study were to evaluate a novel histological grading for hepatoid perianal tumors, single and non-metastatic at presentation, capable of predicting prognosis, and of investigating the Ki67 index as an additional prognostic factor.

## 2 Materials and methods

### 2.1 Case selection and inclusion/exclusion criteria

The cases studied were retrieved from dogs treated at the Veterinary Teaching Hospital (VTH) of the Department of Veterinary Sciences of the University of Turin (Italy) between January 2006 and December 2021, confirmed to be a hepatoid gland tumor based on the histology performed on the surgical specimens. At the time of admission, informed consent was obtained from the dogs’ owners for both the clinical and surgical procedures and the potential use of the results for clinical research studies.

The study included only those dogs with a hepatoid gland tumor, clinically single at presentation that was surgically excised, and staged T1-T3/N0/M0 (11); in addition, complete survey data and a tissue specimen of adequate quality had to be available. Therefore, the exclusion criteria included the presence of distant metastases, an incomplete clinical report and a follow-up time of less than 6 months. Dogs clinically bearing multiple hepatoid nodules or those with sublumbar lymphadenopathy at admission were also excluded.

### 2.2 Histopathology

Archived paraffin-embedded tissues were retrieved, sectioned (4 μm), and stained with hematoxylin and eosin (HE). The histological slides were reviewed independently by 2 pathologists (L.M. and S.I.). They were blinded to the original histological diagnosis, clinicopathological data and clinical outcome; all the discordant results were reviewed by the 2 pathologists with a multiheaded microscope to reach a consensus based on the criteria established by the latest classification (19).

The tumor features were evaluated for all cases, regardless of diagnosis. To standardize the assessment, numerical values were assigned to the characteristics shown in Table 1. The mitotic count was assessed in areas of 2.37 mm2 for all cases, searching for the zones of the highest proliferative activity, which were then expressed as an arithmetic average of the counts reported by the two pathologists (20). Finally, the values were reported in an Excel chart (Microsoft® Excel® 2016 MSO.).

**Figure 1:**
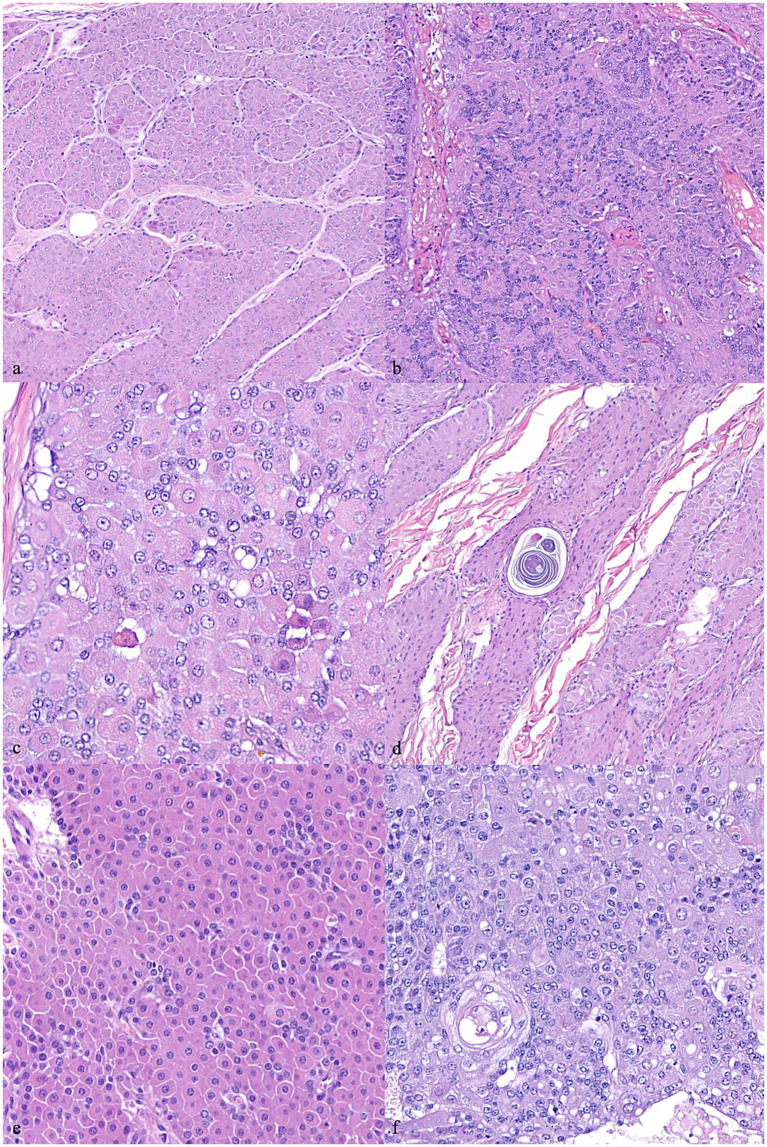
Canine hepatoid gland tumors. **(A)** Presence of trabecular/lobular architecture. 200x magnification. **(B)** Loss of trabecular/lobular architecture. 200x magnification. **(C)** Sebaceous differentiation. 400x magnification. **(D)** Ductal differentiation. 400x magnification. **(E)** Cellular pleomorphism assessed as normal – score 0. 400x magnification. **(F)** Cellular pleomorphism assessed as severe – score 2. 400x magnification. Hematoxylin and eosin staining.

**Table 1:** Tumor features evaluated.

	Evaluation		
Capsule or pseudocapsule	Absent	Present	/
Calcific or mineralized intralesional foci	Absent	Present	/
Ulceration	Absent	Present	/
Loss of lobular/trabecular architecture	No	Yes (more than 50%)	/
Sebaceous differentiation	Absent	Present	/
Ductal differentiation	Absent	Present	/
Tumor infiltrating lymphocytes	Absent	Present	/
Infiltrative growth	Absent	Present	/
Multinucleated cells	Absent	Present	/
Cellular pleomorphism	Regular, uniform cells having small nuclei with occasional small nucleoli	Moderate degree of variation in nuclear size and shape with nucleoli which can be prominent	Notable variation in nuclear size and more

### 2.3 Establishment of histological grading

A novel grading system was created for hepatoid gland tumors using prognostic histological characteristics, such as weighted sums. This indicated that all the categorical features (given as binary values, 0/1) were summed up together after being weighted by their hazard ratios which were derived from a survival regression model measuring their association with time to recurrence. The grading was created as follows:

$$\mathrm{Grading}\left(x\right)=\{\mathrm{high},\;\underset{i}{\sum }Hz_{i}x_{i}>10\;\mathrm{low},\;\underset{i}{\sum }Hz_{i}x_{i}\leq 10$$

where x is the list of the canine patient’s characteristics and Hz is the relative hazard ratio.

### 2.4 Ki67 immunohistochemical staining and automated evaluation

Immunohistochemical staining for Ki-67 was carried out on additional sections obtained from formalin fixed paraffin embedded (FFPE) samples. Briefly, four μm sections of FFPE tissue were cut, mounted on positively charged slices (Menzel-Gläser Superfrost plus. Thermo scientific), deparaffinized in xylene, rehydrated in graded ethanol and rinsed in distilled water. Endogenous peroxidases were blocked by incubating sections for 20 min in 0.3% hydrogen peroxide diluted in distilled water.

Antigens retrieval was performed placing sections in a microwave-resistant container with a solution of tris-EDTA (pH 9.0) and heating 3 times (3 min each) at 700 W. The sections were left to cool at room temperature for 15 min and were next immersed in phosphate buffer saline solution (pH 7.4) for 5 min.

Tissue sections were subsequently incubated with Blocking serum (Vector® Vectastain Elite ABC kit) for 20 min in a humidified chamber, then incubated with the monoclonal mouse anti-human Ki-67 antibody (clone MIB-1, M7240, Dako, Glostrup, Denmark) diluted 1:200 in a PBS solution, in a humidified chamber for 120 min.

After incubation, tissue sections were washed in PBS-tween20 diluted 1:1000 and then incubated with biotinylated secondary antibody (Vector® Vecstain Elite ABC kit, Anti-Mouse IgG/Rabbit IgG) in a humidified chamber, for 30 min.

Avidin-biotin system (Vecstain®) was used for immunolabeling, and reactions were visualized with 3′3′-diaminobenzidine (DAB, Peroxidase Substrate Cat. No. SK-4100). QuPath software was used for assessing the Ki67, following the guidelines provided by Pai et al. (21) and checking step by step that the cell selection provided by the software was correct (21). Briefly, for each case prepared, 3 fields were evaluated from Ki67-labeled slides in Ki67 hotspots (areas of the greatest immunolabeling of the tumor cells for this proliferation marker). The fields were photographed at 20x with a camera (Leica ICC50, Leica®, Germany), placed on an optical microscope (Leica DM750, Leica® Germany) and acquired as images on a personal computer using dedicated software (Leica Application Suite X 5.1.0.25593, Leica®, Germany). The settings used are summarized in Supplementary File S1.

### 2.5 Follow-up

The owners or referring veterinarians were contacted to update the follow-up of the dogs included in the study to ascertain whether the tumor had recurred, whether the dog was still alive, or had died and the cause of death. Overall survival (OS) was considered as the number of days from surgery to death, while the disease-free interval (DFI) was considered as the number of days from surgery to tumor recurrence, and/or evidence of regional (cytologically confirmed) or distant metastasis. The cases were censored using right censoring (22, 23).

### 2.6 Statistical analysis

A grid search was used to identify the cut-off values to apply when using clinical prediction models to assign triage levels to the canine patients. This was carried out using a grid search to optimize the difference between both the median estimated OS and the DFI using the data uncovered by the newly found cut-offs. After having set an appropriate cut-off point for each continuous feature in order to reduce the degrees of freedom of the dataset, the prognostic relevance of each feature was accessed using a univariate regression model (survreg). The features having a *p*-value under the chosen statistical threshold of 0.05 were acknowledged to be independently relevant, requiring additional analysis.

The statistically significant parameters emerging from the univariate analysis regarding DFI were used to generate a scoring system. This score was defined as the sum of the hazard ratios associated with the presence of each characteristic. Finally, a suitable cut-off approximation was carried out regarding the newly designed score to divide (non-censored) canine patients into two categories (good and bad prognosis). The cut-off was then validated on both OS and DFI using the regression model. Clinical and pathological results were grouped into contingency tables and analyzed using Fisher’s exact or Chi square tests. All the analyses were carried out in an R environment (R Software v.4.3.1).

## 3 Results

A total of 76 hepatoid perianal gland tumors were retrieved from an equal number of dogs. Of the canine population examined, 66 were male (85.0%) of which 11 were neutered (16.6%), and 10 were female (15.0%) of which 6 were spayed (6.0%). There were 30 mixed-breed dogs (39.0%) and 46 purebred dogs (61.0%). The mean age was 11.4 years (SD ± 2.4 years). The mean weight was 18.4 kg (SD ± 13.4 kg). Regarding the surgical procedure, tumor excision with a 5–10 mm margin was attempted in all cases and was combined with castration in 45 cases (59.2%). In 31 dogs (40.8%), the tumor excision was not combined with neutering; 10 intact male dogs out of 55 (18.0%) were not castrated at owners’ request. The mean tumor size was 2.5 cm (SD ± 2.2 cm). Of the 45 dogs which underwent castration, histopathology revealed a testicular neoplasm in 18 dogs (40%). In particular, 9 dogs (50.0%) had a seminoma, 2 dogs (11.0%) a seminoma and a Leydig cell tumor, 1 dog (5.5%) a seminoma and a Sertoli cell tumor, 3 dogs (17.0%) a Sertoli cell tumor, 1 dog (5.5%) a Sertoli cell tumor and a Leydig cell tumor, and 2 dogs (11.0%) a Leydig cell tumor only. The perianal tumors were histologically classified as adenomas (19 cases, 25.0%), adenocarcinomas (50 cases, 65.8%) and epitheliomas (7 cases, 9.2%). The median mitotic count was 9.27 ± 10.49. The hepatoid tumors were histologically completely resected in all cases. The overall recurrence rate was 13%. Among the 10 dogs that showed signs of local recurrence, all were diagnosed with adenocarcinoma, and they included 1 female (10%), 2 neutered males (20%), and 7 intact males (70%); the latter had all been neutered during the excision of the hepatoid tumor. The metastatic rate was 9.2%; metastases developed in the regional sacral lymph nodes in 7 cases, lungs in 2 cases and liver in 1 case. The dogs with metastases were 2 spayed female and 5 male dogs neutered at the time of the tumor excision. Fifty-four of 76 cases were censored as these dogs died from tumor-unrelated causes or were lost during the follow-up period. The median overall DFI and OS was 730 days. The clinical and histological results were compared with the follow-up data and are summarized in Table 2.

**Table 2:** Clinical and histological results in comparison with the follow up data.

Variable		Median DFI (days)	Median OS (days)	*p* value DFI	*p* value OS
Sex	Male	303	498	*p* > 0.05	*p* > 0.05
	Female	1963	1981		
	Neutered male	/	899		
	Spayed female	345	498		
Weight	≤7 kg	254	570	*p* > 0.05	*p* > 0.05
	>7 kg	1963	1,542		
Tumor size	T1	1963	1,460	*p* > 0.05	*p* > 0.05
	T2	630	1,121		
	T3	287	662		
Testicular tumors	Absent	700	899	*p* > 0.05	*p* > 0.05
	Present	303	–		
Mitotic count	≤11	1963	1,542	*p* = 0.003*	*p* > 0.05
	>11	303	735		
Multinucleated cells	Absent	1963	1,542	*p* = 0.002*	*p* > 0.05
	Present	303	498		
Loss of lobular/trabecular architecture	No (Figure 1A)	1963	1981	*p* = 0.0009*	*p* = 0.003*
	Yes (Figure 1B)	324	570		
Sebaceous differentiation	Absent	1963	1,542	*p* = 0.02*	*p* > 0.05
	Present (Figure 1C)	345	635		
Calcific foci	Absent	700	1,460	*p*> 0.05	*p* > 0.05
	Present	630	899		
Infiltrative growth	Absent	1963	1,542	*p* > 0.05	*p* > 0.05
	Present	324	700		
Ductal differentiation	Absent (Figure 1D)	1963	1,542	*p* = 0.015*	*p* = 0.04*
	Present	324	570		
Cellular pleomorphism	Regular (Figure 1E)	2045	1,246	*p* = 0.002*	*p* = 0.01*
	Moderate	1,286	617		
	Severe (Figure 1F)	1,081	351		

*statistically significant association.

The histological features were recorded and analyzed, considering those parameters which better correlated with prognosis (Supplementary File S2). Such features included anisocytosis, anisokaryosis, mitotic count, presence of tumor-infiltrating lymphocytes (TILs), sebaceous differentiation (the presence of cytoplasmic lipidisation), existence of a capsule, calcifications, infiltrative growth, ductal differentiation (squamous metaplasia), loss of lobular/trabecular architecture, ulceration and multinucleated cells.

A grading system was assessed including low and high grade (Table 3) based on whether they were associated with low or high probability of having a worse prognosis, respectively, and was applied by the two pathologists (L.M.; S.I.).

**Table 3:** Schematic summary of the new grading model proposed in this study.

Histological feature	Score	*p* value DFI	*p* value OS
Trabecular/lobular architecture	3.02: absence 0: presence	*p* = 0.0009*	*p* = 0.003*
Multinucleated cells	0: absence 2.27: presence	*p* = 0.002*	*p* > 0.05
Cellular pleomorphism	0: regular 2.78: moderate 5.56: severe	*p* = 0.002*	*p* = 0.01*
Mitotic count	0: 0–7 2.21: >7	*p* = 0.003*	*p* > 0.05
Sebaceous differentiation	0: absence 1.92: presence	*p* = 0.02*	*p* > 0.05
Ductal differentiation	0: absence 2.48: presence	*p* = 0.015*	*p* = 0.04*
Total score:	≤ 10: low grade >10: high grade	*p* = 0.003	*p* = 0.022

Each parameter has been correlated with the survival data. *statistically significant association.

**Figure 2:**
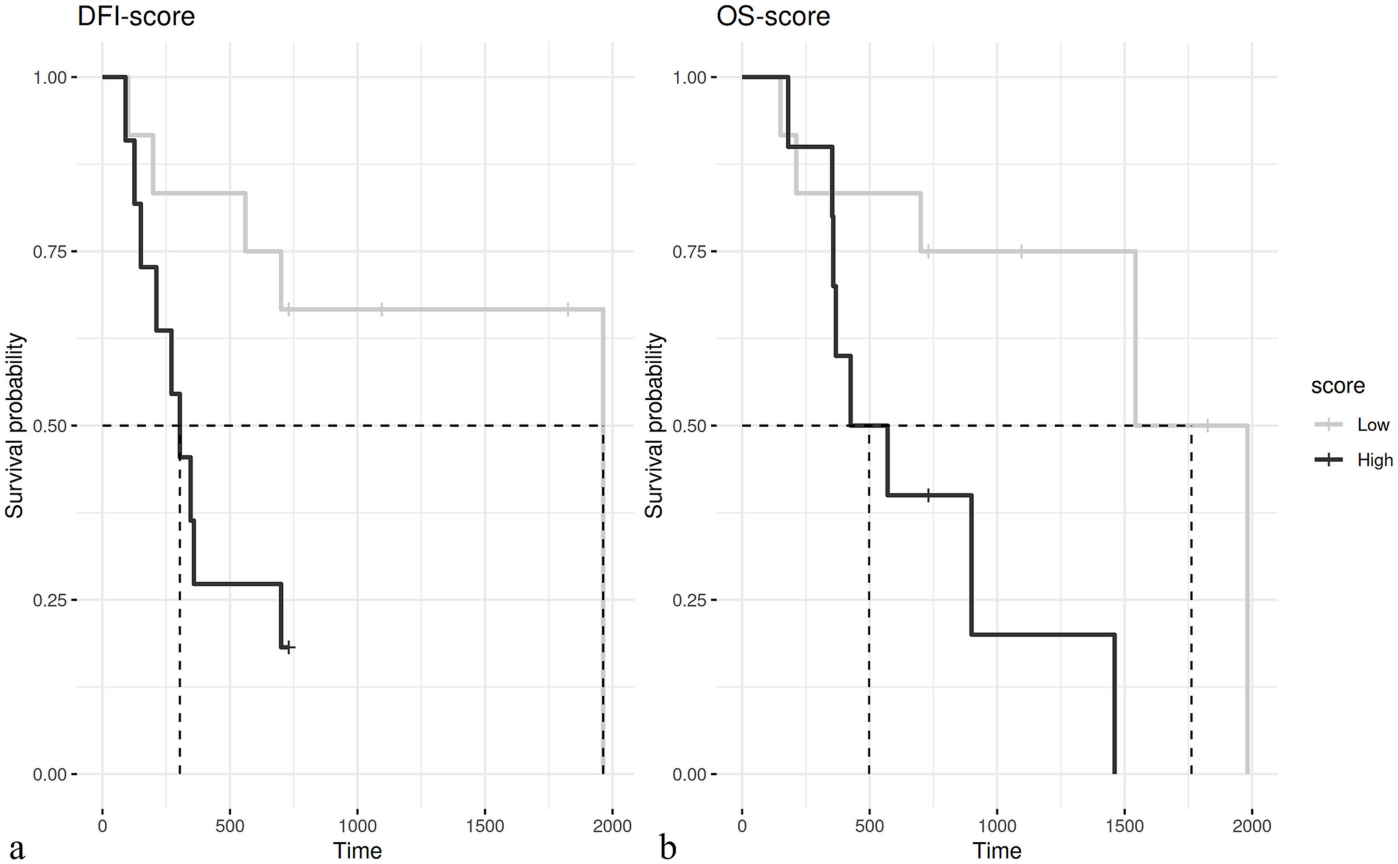
**(A)** Kaplan–Meier curve of the disease-free interval (DFI) in dogs with low grade (median 1963 days) and high grade (median 303 days – *p* = 0.003) tumors. **(B)** Kaplan–Meier curve of overall survival (OS) in dogs with low grade (mean 1762 days) and high grade (mean 498 days – *p* = 0.02) tumors.

According to this novel histological grading, a total of 38/76 cases were classified as low grade (50%) and 38/76 cases as high grade (50%) malignancy. Regarding the original diagnosis, of the cases with a previous diagnosis of adenoma (19 cases), according to the grading system presented herein, the majority were classified as low grade (18/19), with only 1 case being a high grade, based on the grading system proposed herein. The epitheliomas were classified as low grade in 2/7 cases and high grade in 5/7 cases, while the adenocarcinomas were classified as low grade in 18/50 cases and high grade in 32/50 cases. According to this novel grading system, the histological grade was strongly associated with the recurrence (Chi-square test; *p* = 0.003). In particular, three out of 38 dogs (7.9%) with adenocarcinomas classified as low grade and 7 out of 38 (18.4%) dogs with tumors classified as high grade developed a local recurrence. Metastases were confirmed in 3 out of 38 (7.9%) cases with adenocarcinomas classified as low grade and in 4 out of 38 (10.5%) cases classified as high grade. The histological grade was also associated with the patients’ outcome (*p* = 0.003 for DFI and a *p* = 0.02 for OS); in particular, a median DFI of 1963 days and a median OS of 1762 days was recorded in dogs bearing a low grade hepatoid gland tumor as compared with a median DFI of 303 days and a median OS of 498 days in those dogs bearing a high-grade tumor (Figure 2). The histological grades in relation to clinicopathological data are summarized in Table 4. In addition, histological grade was applied only to malignant tumors showing it was associated with relapse (*p* = 0.04) but not with survival.

**Table 4:** Histological grade in relation to the clinicopathological data.

Feature		Low grade (n)	High grade (n)	*p*-value
Sex	M F MC FC	25 3 7 3	30 1 4 3	0.52
Tumor size	T1 T2 T3	25 10 3	21 11 6	0.56
Testicular tumor	Absent Present	18 7	19 11	0.57
Recurrence	Absent Present	35 3	31 7	0.30
Metastasis	Absent Present	35 3	34 4	1
Ki67 index	≤6 >6	26 11	24 14	0.62

**Figure 3:**
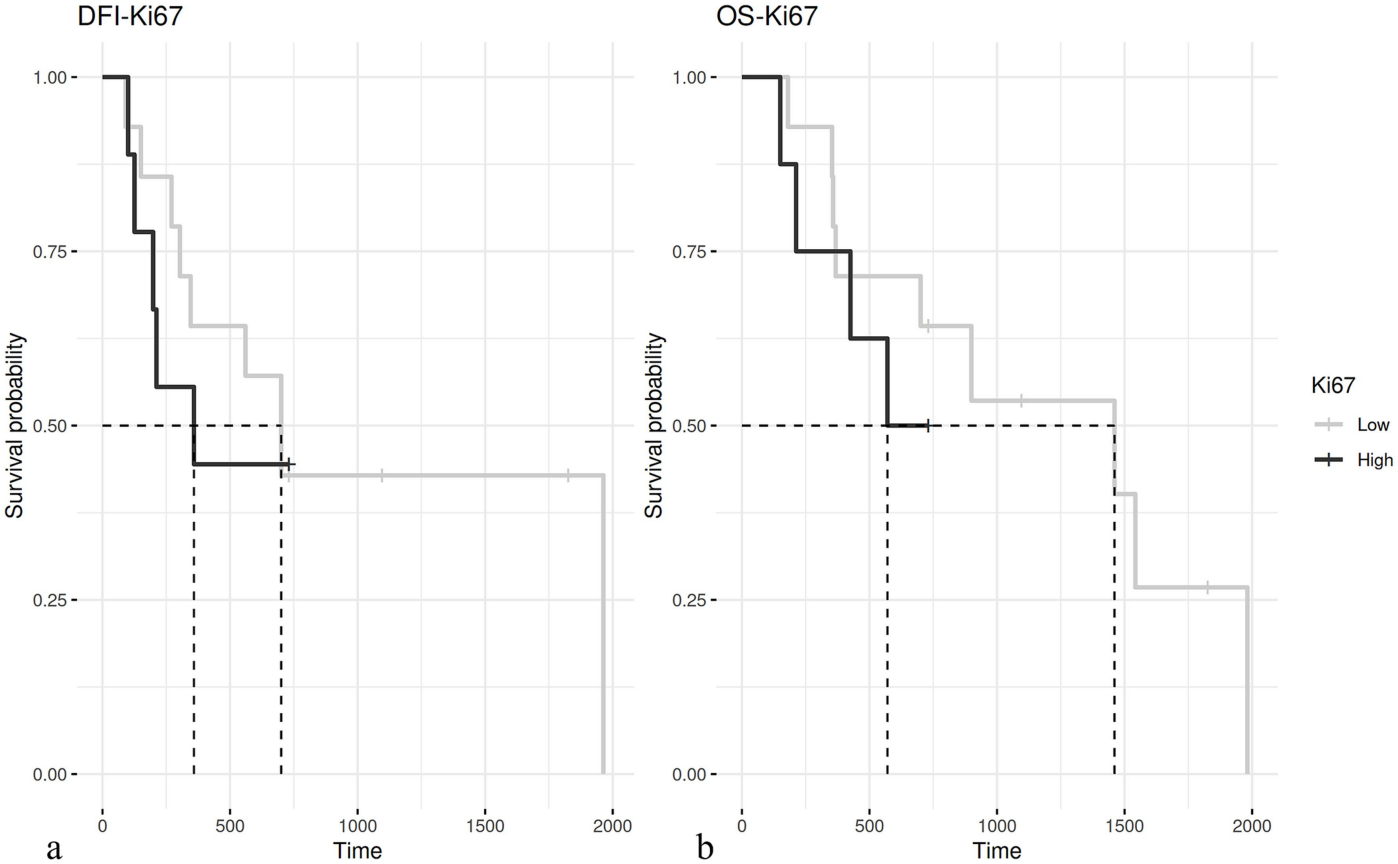
**(A)** Kaplan–Meier curve of disease-free interval (DFI) in dogs having hepatoid gland tumors with a Ki67 ≤ 6% (median 700 days) and > 6% (median 358 days – *p* = 0.5). **(B)** Kaplan–Meier curve of overall survival (OS) in dogs with hepatoid gland tumor with a Ki67 ≤ 6% (median 1,460 days) and > 6% (median 570 days – *p* = 0.36).

The evaluation of the Ki67 index was available for 75 out of 76 cases; the median value was 4.84. With the cut-off set at 6%, the Ki67 index was not correlated with prognosis (Figure 3).

## 4 Discussion

In the cohort of cases presented here, the dogs were mainly mixed breeds (39.9%) with a mean age of 11.4 years (SD ± 2.4 years), which, according to the literature (19, 24), is slightly higher than previously reported (3, 25, 26). The clinical features of the hepatoid tumors that were seen in this study mirror those reported in the literature, i.e., perianal tumors, well circumscribed in most cases, having an average diameter between 0.5 and 3 cm (3). Most of the dogs of this study were intact males (85%), and this finding correlates to the development of most low-grade hepatoid tumors with a hormonal influence (3, 4). In particular, it is known that androgenic hormones stimulate the growth of the hepatoid gland cells expressing the receptors. It is also known from the literature that castration alone may induce a substantial cytoreduction of most hepatoid adenomas (27). In intact male dogs, a correlation between perianal adenomas and testicular interstitial cell tumors has been suggested and this support a testosterone stimulation as a causative factor (4, 5). Even in the present cohort of cases, 40.0% of the dogs had a concurrent testicular tumor. However, only 27.5% of these dogs had an interstitial cell tumor and 50% had a seminoma, bearing the remaining a Sertoli cell tumor, or both a Sertoli cell tumor and a seminoma. This phenomenon may indicate that a more complex hormonal change may play a pathogenetic role (28). At present, the standard of care for perianal adenomas is marginal excision associated with castration; conversely, perianal adenocarcinoma should be addressed by a more aggressive surgery, with a minimum margin of 1–2 cm (3, 29). The prognosis for adenocarcinomas seems related to the clinical tumor stage and the completeness of the surgical excision (13). Due to the anatomic contiguity between most of these hepatoid tumors and the external anal sphincter muscle, a wide margin excision is rarely performed in an attempt to preserve fecal continence. Nevertheless, in most of the cases presented in this retrospective study the histologic evaluation of the excision margins indicated a complete surgical excision. Overall, the recurrence rate was 13%, that is comparable to that reported in the literature (2). Currently, the guidelines for the classification of epithelial neoplasms generically divide hepatoid gland tumors into adenoma, epithelioma and adenocarcinoma (without referring to different degrees of differentiation) (15). According to the literature, hepatoid gland adenocarcinomas are characterized by a low rate of both recurrence and metastasis (3, 12); however, to date, a histological grading system capable of identifying cases with a worse prognosis is missing. The literature has reported that a few hepatoid tumors with well-differentiated morphology, in the absence of any clinical tumor staging demonstrating the presence of metastases, would have been diagnosed as adenoma based on cell morphology alone (16); this finding was also confirmed by the present study (unpublished data) and by one case of the present cohort of dogs which developed a metastasis after 198 days, despite a hepatoid tumor with histological features compatible with a benign lesion.

The histological grade proposed in this study was designed to be applicable to all hepatoid neoplasms, either diagnosed as adenomas, epitheliomas, or adenocarcinomas, so that their prognosis could be predicted. The histological parameters that were found to be useful in constructing this histological grading system are largely those that have previously been identified as carcinoma-related.

Although this grading system offers better assistance in differentiating low-grade from high-grade hepatoid neoplasms, the existence of a small cohort of well-differentiated tumors which may develop metastases needs further consideration. The Ki67 index has been used as a prognostic indicator for some tumors in various studies (30–34); however, the different results that are often obtained may derive from the different immunohistochemical techniques and evaluation methods used. In the present study, the Ki67 index was evaluated using the QuPath software to strongly limit these evaluation bias, but it was not found to be associated with prognosis anyway. This result differs from the study by Pereira et al. (14) who, using a computer-assisted image analysis, found that Ki67 with a cut-off assessed at 9.87% was predictive of recurrence. Therefore, although for many canine cancers, Ki67 seems to be a valuable prognostic factor (30–34), this was not the case for the hepatoid tumors presented in this case cohort. Nonetheless, the Authors encourage its use as an additional prognostic information since a single case diagnosed as adenoma and classified as low-grade by the present study had a Ki67 of 25.37% which was considerably above the cut-off considered by both the authors of this study and Pereira et al. and developed metastasis after 198 days, pointing out that, in selected cases, Ki67 can provide useful prognostic information.

Even if this study has two main limitations due to its retrospective nature and the limited number of cases included, it opens the way to a grading system which may be considered as an important and valid diagnostic tool for formulating a more accurate diagnosis and prognosis of canine hepatoid tumors. Nevertheless, further studies recruiting a larger number of cases are warranted in order to confirm these preliminary results.

## 5 Conclusion

The histological grading of hepatoid perianal tumors remains a challenge for the veterinary pathologists. Despite the fact that the prognosis for these tumors is often favorable following surgical excision, the Authors emphasize the importance of the collaboration between clinicians and pathologists in the evaluation of the clinical tumor staging, the long-term follow-up, and the histological appearance of these tumors. The Authors believe that the use of the histological scoring system proposed in this study may potentially help the understanding of the clinic-biological behavior of these tumors as additional prognostic information is added; at the same time, the Authors feel that further studies are warranted to confirm these preliminary data.

## Data Availability

The raw data supporting the conclusions of this article will be made available by the authors, without undue reservation.
