# Supplementary material for: A novel scoring system proposal to guide treatment of dogs with hepatoid gland tumors
Source: Front Vet Sci. 2025 Feb 5;12:1451510. doi: 10.3389/fvets.2025.1451510 (PMC11841504; doi:10.3389/fvets.2025.1451510)
Supplement: Supplementary file 1 [file Table_1.DOCX]

Supplementary Material

# Supplementary Data S1

# The images were stored as .tiff files and imported into QuPath 0.2.3 with the extension "Heme/DAB brightfield". The image files were then analyzed to approximate a count of at least 500 tumor cells in Ki67 hotspots (areas of highest tumor cell positivity for this proliferation marker). The settings for magnification and resolution were kept constant. Optimal settings were determined by "trial and error" and included the following: photomicrograph at 20×, resolution of 2080 × 1536 pixels, with 50% zoom, staining vectors with RGB pixel depth (red, green, blue) were recalibrated with "Estimate Stain Vectors" before algorithm counting using the default "auto" detection. The heme threshold for counterstaining "threshold" was changed from the default setting (0.10) to 0.001; "threshold Compartment" was set to "Nucleus: DAB OD mean" with "single Threshold" set to "true"; "threshold Positive1" was set to "0.08).
